# Initial experience with single-port robot-assisted partial nephrectomy using the Low Anterior Access (LAA): perioperative outcomes and learning curve analysis in a Belgian SP-naive tertiary robotic referral center

**DOI:** 10.3389/fsurg.2025.1734877

**Published:** 2026-01-07

**Authors:** Henri Van Eecke, Julien Grammens, Peter De Kuyper, Wesley Verla, Filip Ameye, Simone Crivellaro, Karel Decaestecker

**Affiliations:** 1 Department of Urology, Maria Middelares Hospital, Ghent, Belgium; 2 Department of Urology, Catholic University Leuven, Leuven, Belgium; 3 Faculty of Medicine and Health Sciences, Department of Human Structure and Repair, Ghent University, Ghent, Belgium; 4 Department of Urology, University of Illinois at Chicago, Chicago, IL, United States

**Keywords:** learning curve, minimally invasive techniques, partial nephrectomy, perioperative outcomes, retroperitoneal, robot-assisted surgery, single-port robot

## Abstract

**Objectives:**

Robot-assisted partial nephrectomy (RAPN) has become a standard minimally invasive approach for localized renal tumors. The introduction of the single-port (SP) robotic system has enabled novel retroperitoneal techniques, such as the Low Anterior Access (LAA). This study presents the initial experience of SP RAPN using the LAA technique in a SP-naive Belgian tertiary robotic referral center.

**Methods:**

A retrospective database was created with prospectively collected data from patients who underwent SP RAPN via LAA between May 2024 and September 2025. All procedures were performed by a SP robot-naive surgeon with extensive multi-port transperitoneal robotic experience but no prior multi-port retroperitoneal exposure. Surgical and perioperative outcomes of SP RAPN, using the LAA technique, were the primary endpoint of this study. As a secondary endpoint we evaluated the learning curve of this procedure.

**Results:**

Forty patients underwent SP RAPN. Median tumor size was 26 mm, with a median RENAL score of 5. Median warm ischemia time was 17 min and median estimated blood loss was 50 ml. No intraoperative complications, conversions, or positive surgical margins occurred. Three minor postoperative complications (7.5%) were recorded, with no grade ≥III events. Median length of stay was one night and median postoperative pain scores (VAS) at 12 and 24 hours were 0. Operative time, warm ischemia time and estimated blood loss showed improvement over successive cases, reflecting increased procedural efficiency.

**Conclusion:**

SP RAPN using the LAA technique is feasible and safe, even for a surgeon without prior SP or retroperitoneal experience. This first experience study demonstrated low morbidity, minimal postoperative pain, and early discharge. These findings support the adoption of SP RAPN via LAA as a viable option for minimally invasive nephron-sparing surgery, warranting validation in larger multicenter studies.

## 1 Introduction

For the treatment of localized kidney cancer multiple studies have demonstrated that conventional laparoscopy and robot-assisted laparoscopy result in reduced pain perception, faster recovery, and shorter hospitalization compared to open surgery (1). For partial nephrectomy (PN), it has been shown that robot-assisted laparoscopy, compared to conventional laparoscopy, leads to fewer complications, fewer positive surgical margins, and better preservation of kidney function (2). Today, the Multi-Port (MP) robotic transperitoneal approach is the predominant method to perform a robot-assisted partial nephrectomy (RAPN) (3). The MP retroperitoneal approach provides an alternative technique offering unique advantages, such as avoiding the mobilization of bowel and reduction of postoperative ileus, but also certain disadvantages, in particular clashing of instruments in the small retroperitoneal space. The latter, in the past, has led to a rather poor adoption of the MP retroperitoneal approach by urologists (4, 5).

In 2018, Intuitive Surgical introduced the first Single-Port robotic platform to the market (6). This SP robotic system theoretically offers several advantages compared to the MP system, such as fewer incisions, faster recovery, better cosmetic outcomes and less pain (7). Initial experience has demonstrated that SP RAPN is a safe and feasible procedure, but with slightly higher warm ischemia time and transfusion rate, when compared to MP RAPN (1, 8). On the other hand, SP RAPN has been suggested to result in a shorter hospital stay, lower postoperative pain score and lower inpatient opioid use (8–10). Since the introduction of the SP-robot, there has been a growing trend toward the adoption of the retroperitoneal approach. This shift is largely attributable to the enhanced maneuverability of the SP system within confined anatomical spaces and its facilitated access to the target anatomy (4). For SP RAPN, Pellegrino et al. published their experience with a novel retroperitoneal approach, namely the Supine Anterior Retroperitoneal Access (SARA) or Low Anterior Access (LAA) and demonstrated this surgical technique to be feasible and safe. Moreover, they showed low complication rates, short hospitalization and reduced postoperative pain by using this technique (7). Very recently, Bae et al. published their initial experience with the LAA technique and demonstrated low complication rates and rapid recovery (11).

In 2024, following CE certification, the SP robot was introduced in Europe, with Maria Middelares Hospital (Ghent) becoming the first hospital in Belgium to implement this new robotic technology. To start performing SP RAPN in our center, we decided to implement the LAA technique from the beginning. To evaluate the feasibility of adopting this surgical method we collected the surgical and perioperative outcomes of all retroperitoneal SP RAPN performed at our center.

## 2 Materials and methods

We collected baseline clinical and demographic information and perioperative data of patients who underwent SP RAPN from May 2024 to September 2025 at our institution. Institutional ethical review board approval was obtained prior to data collection (MMS.2024/078).

Patients were eligible for inclusion if they were diagnosed with a cT1a/1b N0 M0 renal mass and underwent a retroperitoneal SP RAPN, using the LAA technique. All procedures were performed by a single urologist (K.D.) with extensive experience in MP robotic surgery (1608 MP procedures including 524 RAPN). Nevertheless, this surgeon had no previous experience with the SP robot, nor with the retroperitoneal approach for performing RAPN. The surgeon only had experience with pure retroperitoneoscopic radical nephrectomy >10 years ago. In the 3 months preceding and including the first clinical cases, the surgeon followed a structured training pathway of case-observation, lab training (cadaveric and porcine models) and proctoring, all guided by experienced SP surgeons using the LAA technique. Prior to surgery, decisions regarding the diagnosis and indication for RAPN were made at multidisciplinary oncological meetings and patients were comprehensively counseled concerning the use of the novel SP platform. The decision to use the SP robotic system for performing the RAPN was made by the treating surgeon and based on the patient's and tumor's preoperative characteristics.

RENAL (Radius, Endophytic/exophytic, Nearness, Anterior/posterior, Location) nephrometry scores were calculated according to the preoperative cross-sectional imaging and radiology reports. We used the Clavien-Dindo classification system for reporting complications (12). The LAA technique used for performing the SP RAPN was analogous to the procedure described by Pellegrino et al. (7). All procedures were performed using a pure SP approach without the use of a site-car port or a plus-one assistant port. A nasogastric tube was used for aspiration. Standard robotic instruments were employed, including scissors, Maryland dissector, fenestrated bipolar or force bipolar, needle driver and robotic clip applier. Scanlan Bulldog clamps were utilized for renal artery occlusion. The inner renorrhaphy was performed using a running 3-0 Monocryl suture, while the outer renorrhaphy was completed with interrupted 2-0 Vicryl sutures. The camera was generally positioned below the working instruments. However, for anterior upper pole tumors, a camera-above configuration was adopted to optimize visualization.

Follow-up of patients consisted of the standard periodic follow-up after RAPN. During hospitalization, pain assessment was performed using the Visual Analogue Scale (VAS) at 12–24 h after surgery.

As a primary endpoint, we investigated the surgical and perioperative outcomes of SP RAPN using the LAA technique. The trifecta of outcomes (negative surgical margins, warm ischemia time <30 min, and Clavien–Dindo grade <3) was evaluated to appraise the quality of RAPN. As a secondary endpoint, we assessed the learning curve to determine the feasibility for an SP-naive robotic surgeon, with no prior clinical experience in the retroperitoneal approach for RAPN, to adopt this procedure. The learning curve was evaluated through analysis of operative time, WIT, and EBL. The cohort was stratified into two groups: the initial 20 cases (early phase) and the subsequent 20 cases (late phase). The distribution of continuous variables was assessed using the Shapiro–Wilk test in conjunction with visual inspection of histograms and Q–Q plots. As none of the variables demonstrated a normal distribution, intergroup comparisons were conducted using the Mann–Whitney *U* test. Continuous data are expressed as median values with interquartile ranges (IQRs) and statistical significance was defined as a *p*-value < 0.05.

We used descriptive statistics to summarize and report the baseline characteristics and perioperative outcomes of our patient cohort. Statistical analyses to assess the learning curve were performed using IBM SPSS Statistics, version 31.0.0.0 (IBM Corp., Armonk, NY, USA).

## 3 Results

Table 1 shows the baseline characteristics of our cohort, including 40 patients (20 males, 20 females), with a median age of 67 (IQR 61–78) years and a median Body Mass Index (BMI) of 27 (IQR 23–29). The majority of our patients had undergone previous abdominal surgery (58%) and 14 patients (35%) had an American Society of Anesthesiologists (ASA) score of 3. Most renal masses were classified as cT1a (85%) and a minority as cT1b (15%). Median tumor size was 26 mm (IQR 20–37) with a median RENAL nephrometry score of 5 (IQR 4–6) and predominance (75%) of low-risk (RENAL score 4–6) tumors. Apart from a slightly higher proportion of interpolar tumors, tumor locations were evenly distributed among patients.

**Table 1:** Baseline patient characteristics.

Patient characteristics	*n* (%) or median (IQR)
*N* (%)	40 (100%)
Age (yr)^a^	67 (61–78)
Sex, *n* (%)	
Male	20 (50%)
Female	20 (50%)
BMI^a^	27 (23–29)
ASA score^a^, *n* (%)	2 (2–3)
I	3 (7,5%)
II	23 (58%)
III	14 (35%)
Preop. eGFR^a^ (ml/min/1.73 m^2^)	77 (58–90)
History of abdominal surgery, *n* (%)	
Yes	23 (58%)
No	17 (43%)
Diagnosis, *n* (%)	
Incidentaloma	37 (93%)
Hematuria	0 (0%)
Loin pain	1 (3%)
Other	2 (5%): recurrent pyelonephritis, recurrence after radiofrequent ablation therapy
Tumor characteristics	*n* (%) or median (IQR)
cT stage, *n* (%)	
cT1a	34 (85%)
cT1b	6 (15%)
Tumor size (mm)^a^	26 (20–37)
RENAL nephrometry score^a^, *n* (%)	5 (4–6)
4	11 (28%)
5	10 (25%)
6	9 (23%)
7	3 (7.5%)
8	4 (10%)
9	3 (7.5%)
Location, *n* (%)	
Anterior	12 (30%)
Posterior	14 (35%)
Neither	14 (35%)
Site of lesion, *n* (%)	
Upper pole	13 (33%)
Interpolar	19 (48%)
Lower pole	8 (20%)

a Median (IQR).

Tables 2, 3 present our surgical and perioperative outcomes. Operative time was subdivided in skin-to-skin (from skin incision to wound closure), robotic console time (from first instrument manipulation by console surgeon to last) and surgical room time (from operation room entry to exit). Median WIT was 17 min (IQR 10–24) and median EBL was 50 ml (IQR 10–108) with only 3 patients (7.5%) having EBL greater than 200 ml. Of all procedures, 23 (58%) were performed with selective clamping, whereas 4 (10%) were conducted clampless. No intraoperative complications were observed and there was no necessity for conversion to radical nephrectomy or alternative surgical approaches (MP, laparoscopic or open). Additionally, no blood transfusions were required intraoperatively. Final surgical pathology did not reveal any positive surgical margins and showed a predominance of pT1a clear cell renal cell carcinoma.

**Table 2:** Surgical outcomes.

Outcome	*n* (%) or median (IQR)
Operative time (min)	
Skin-to-skin^a^	162 (129–186)
Console time^a^	116 (93–143)
Surgical room time^a^	216 (174–255)
Estimated Blood Loss (ml)^a^, *n* (%)	50 (10–108)
>200 ml	3 (7.5%)
100–200 ml	9 (23%)
<100 ml	28 (70%)
Warm Ischemia time^a^	17 (10–24)
Type of clamping, *n* (%)	
Total	13 (33%)
Selective	23 (58%)
Clampless	4 (10%)
Hemostasis, *n* (%)	
Renorrhaphy	20 (50%)
Hemostatic sealing patch	6 (15%)
Renorraphy + hemostatic sealing patch	11 (28%)
None	3 (7.5%)
Intraoperative complications, *n* (%)	0 (0%)
Need for conversion to radical nephrectomy or open procedure, *n* (%)	0 (0%)
Perioperative transfusion need, *n* (%)	0 (0%)
pT stage, *n* (%)	
pT0 (benign)	12 (30%)
pT1a	24 (60%)
pT1b	2 (5%)
pT2a	0 (0%)
pT2b	0 (0%)
pT3a	2 (5%)
pT3b or higher	0 (0%)
Positive surgical margins, *n* (%)	0 (0%)
Tumor histology, *n* (%)	
Clear cell renal cell carcinoma	19 (48%)
Papillary renal cell carcinoma	7 (18%)
Chromofobe renal cell carcinoma	2 (5%)
Oncocytoma	8 (20%)
Benign cyst	3 (7.5%)
Angiomyolipoma	1 (2.5%)

a Median (IQR).

**Table 3:** Perioperative outcomes.

Outcome	*n* (%) or median (IQR)
Postoperative complications 30 days, *n* (%)	
Clavien-Dindo Grade I	1 (2.5%)
Clavien-Dindo Grade II	2 (5%)
≥Clavien-Dindo Grade III	0 (0%)
Postoperative pain^a^ (VAS)	
12 h postoperatively	0 (0–0)
24 h postoperatively	0 (0–1)
Length of stay^a^ (days), *n* (%)	1 (1–2)
1 day	28 (70%)
2 days	9 (23%)
≥3 days	3 (7.5%)
Readmission rate, *n* (%)	1 (2.5%)

VAS, visual analogue scale.

a Median (IQR).

In this cohort, postoperative complications were observed in 3 patients. One patient developed a retroperitoneal hematoma on postoperative day 1, requiring a single unit blood transfusion and later on intravenous antibiotics for suspected early superinfection of the hematoma. This patient accounted for the 8-day length of stay (LOS) outlier. The other complications were an ascending urinary tract infection and acute urinary retention after catheter removal. No high grade Clavien-Dindo complication (Grade III or higher) were observed. Median both 12- and 24 h postoperative pain scores (VAS) were 0. The majority of patients (70%) were discharged on the first postoperative day, with a single patient being readmitted (2.5%) 1 week post-discharge due to a urinary tract infection.

**Figure 1:**
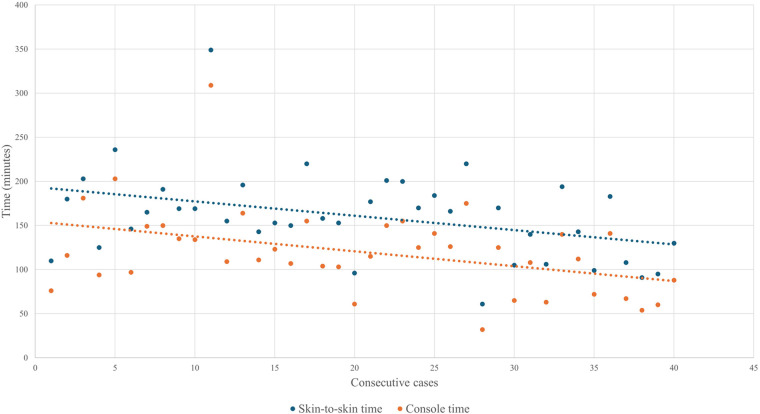
Evolution of skin-to-skin and console time over the first forty cases.

**Figure 2:**
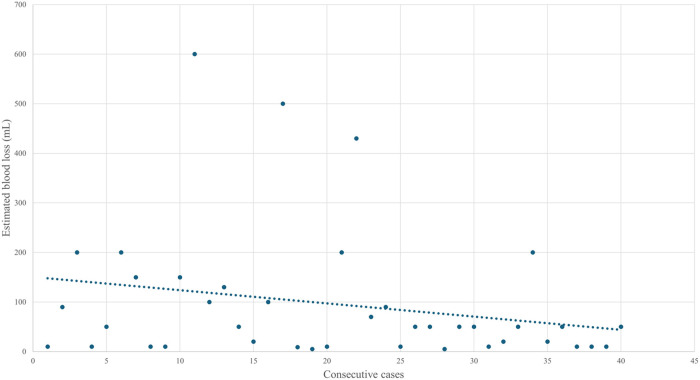
Evolution of estimated blood loss over the first forty cases.

**Figure 3:**
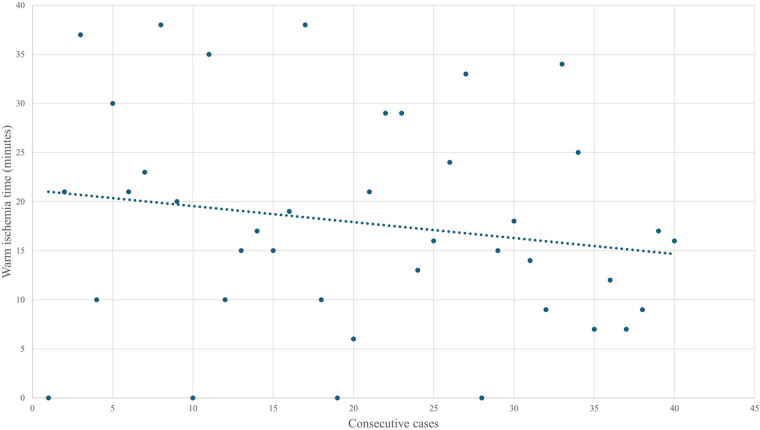
Evolution of warm ischemia time over the first forty cases.

The Mann–Whitney *U* test showed that median surgical room time, skin-to-skin time, console time, WIT, and EBL were lower in the final 20 cases than in the initial 20, although the differences were not statistically significant (Table 4). Given the small sample size, no firm statistical conclusion can be drawn from these findings. As shown in Figure 1, operative and console times tended to decrease with increasing surgical experience, with similar trends observed for EBL and WIT (Figures 2, 3).

**Table 4:** Perioperative outcomes in early versus late phases of the learning curve.

Outcome	First 20 cases (early)	Subsequent 20 cases (late)	*p*-value
Surgical room time^a^ (min)	221 (195–255)	201 (157–252)	0.185
Skin-to-skin time^a^ (min)	162 (147–195)	155 (105–184)	0.234
Console time^a^ (min)	120 (103–154)	114 (66–141)	0.176
Estimated blood loss^a^ (mL)	70 (10–150)	50 (10–65)	0.467
Warm Ischemia Time^a^ (min)	18 (10–28)	16 (10–25)	0.766

a Median (IQR).

## 4 Discussion

Minimally invasive robotic approaches for PN continue to evolve, with an increasing focus on reducing surgical morbidity and improving recovery. In particular, since the introduction of the SP robot, with its articulating instruments and single-incision design, there has been growing interest in expanding the use of retroperitoneal approaches (13). Within this context, the present study describes our early experience with the LAA technique and evaluates its perioperative outcomes in the first forty patients treated.

Despite the lack of prior experience with the SP robot and no previous experience with the robotic retroperitoneal approach for RAPN, the perioperative outcomes of these first forty cases are promising. Overall, our perioperative outcomes are consistent with those published for SP RAPN (9, 14–16). Our results demonstrate a readmission rate of only 2.5%, which is notably lower than the 6% reported by Cannoletta et al. (13). This difference may possibly be explained by the fact that, in their cohort, 85% of patients were discharged on the day of surgery, whereas in our center, discharge generally occurs on postoperative day one. Further investigation is warranted to assess the feasibility and, more importantly, patient acceptance of same-day discharge following SP RAPN within the framework of the Belgian healthcare system.

Postoperative VAS scores were consistently low. Beyond reduced pain levels, we observed an impression of enhanced overall postoperative well-being among patients undergoing this novel technique. Several factors may contribute to this observation. Notably, with the LAA, patients remain in the supine position throughout the procedure, in contrast to the lateral decubitus position typically employed during transperitoneal and MP retroperitoneal techniques. The latter can predispose, particularly in obese individuals, to underrecognized peripheral nerve injuries and related discomfort (17). Furthermore, the avoidance of both pneumoperitoneum and bowel manipulation, inherent advantages of the retroperitoneal route, may further enhance postoperative comfort and recovery. This hypothesis will be addressed in our ongoing prospective study, which incorporates anesthesiological endpoints, such as the Quality of Recovery-15 (QoR-15) questionnaire, to provide a more holistic assessment of postoperative recovery following SP robot-assisted procedures. Very recently, Lambertini et al. reported on the anesthesiologic impact of the LAA technique and demonstrated improved perioperative ventilatory, cardiovascular and pain-related outcomes (18).

Despite this being our initial series of forty cases, no intraoperative complications were encountered, and no conversions to radical nephrectomy or open surgery were required. Moreover, no high grade and only three low grade complications according to the Clavien-Dindo classification were observed during the first postoperative month.

From an oncological point of view, no positive surgical margins were observed. Using the trifecta to assess the quality of PN efficacy (negative surgical margins, WIT <30 min, Clavien-Dindo grade <grade 3), six out of forty patients (15%) did not pass the test (19). More specifically, six patients did not reach the endpoint because of a slightly higher WIT (mean 36 min). However, in this six-patient cohort, four patients underwent surgery during the initial half of the learning curve and the two patients in this cohort operated on during the second half of the learning curve both presented with cT1b tumors, having RENAL scores of 8 and 9, respectively. Moreover, in five out of six patients, the clamping was (super)selective, which decreases the relevance of WIT <30 min.

Recently, Raver et al. reported on the increased utilization of the retroperitoneal approach to perform RAPN when adopting the SP robotic system (4). This observation is particularly evident in our center, where the surgeon involved in this study has performed almost exclusively retroperitoneal SP RAPN since the introduction of the SP platform, having largely abandoned the MP transperitoneal approach except in a few selected cases. During the study period, only seven MP transperitoneal RAPN (7/47 = 15% of total RAPN volume) were performed by this surgeon to treat tumors with high complexity score and/or with anticipated abundant presence of adherent perinephric fat. Whether the SP LAA technique is also feasible in such complex cases remains to be determined and probably depends on the experience of the surgeon.

The exact learning curve and training requirements for a safe transition from MP to SP procedures, and specifically to SP RAPN, have not been clearly defined. Based on our experience, extensive familiarity with robotic surgery in general along with in-depth knowledge of the relevant anatomical structures in the retroperitoneum, appear to result in a shorter and steeper learning curve than initially anticipated. This notion is particularly supported by the encouraging perioperative outcomes observed in this early experience series. Our findings are undoubtedly encouraging for urologists with extensive experience in robotic surgery, considering implementation of the SP-robot and retroperitoneal SP RAPN in their practice. Nevertheless, we recommend that early cases be performed under expert proctorship to ensure safe adoption of the technique and to facilitate management of any unexpected intraoperative challenges.

Several limitations should be acknowledged in this study. First, we conducted a retrospective analysis of prospectively collected patient data. This design may have introduced potential selection bias into our study. Second, the generalizability of our findings may be limited by the relatively small sample size and the single center and single surgeon design, potentially introducing center- and operator-related biases. Third, given its purely descriptive and non-comparative design, this study does not allow for direct evaluation of differences between groups.

## 5 Conclusion

Our initial experience demonstrates that SP RAPN using the LAA technique is both feasible and safe, even for a SP–naive surgeon with limited prior experience in the retroperitoneal approach. The procedure yielded encouraging perioperative outcomes with low complication rates, no positive surgical margins and excellent postoperative recovery profiles. Further prospective, multicenter studies with larger cohorts and longer follow-up are warranted to validate these results, define the learning curve more precisely, and assess the long-term functional and oncological outcomes of this novel approach.

## Data Availability

The original contributions presented in the study are included in the article/Supplementary Material, further inquiries can be directed to the corresponding author.
